# Genetic analyses reveal panmixia in Indian waters and population subdivision across Indian Ocean and Indo-Malay Archipelago for *Decapterus russelli*

**DOI:** 10.1038/s41598-023-49805-8

**Published:** 2023-12-21

**Authors:** Anjaly Jose, Sandhya Sukumaran, Subal Kumar Roul, P. Abdul Azeez, Shoba Joe Kizhakudan, Neenu Raj, K. Nisha, A. Gopalakrishnan

**Affiliations:** 1 Marine Biotechnology Fish Nutrition and Health Division, ICAR-Central Marine Fisheries Research Institute, Ernakulam North P O, Kochi, Kerala 682018 India; 2 Mangalore University, Mangalagangotri, Mangalore, Karnataka 574199 India

**Keywords:** Animal biotechnology, Biotechnology, Genetics, Evolution, Molecular evolution

## Abstract

The Indian Scad, *Decapterus russelli* is an important pelagic carangid widely distributed throughout the Indian Ocean and the Indo-West Pacific. Despite being widely distributed in the Indian Ocean, the information regarding genetic structuring and diversity has been lacking compared to its Indo Malay Archipelago counterparts. The present study was conducted to investigate the genetic stock structure of *D. russelli* based on mitochondrial (*Cyt b*) and nuclear (*DrAldoB1*) markers along Indian waters. The results indicated the presence of a single panmictic stock across the Indian Ocean region. High haplotype diversity associated with low nucleotide diversity suggested a population bottleneck followed by rapid population growth. Phylogenetic analysis revealed the absence of geographical clustering of lineages with the most common haplotype distributed globally. The pelagic life style, migratory capabilities, and larval dispersal may be the contributing factors to the observed spatial homogeneity of *D. russelli*. However, significant genetic differentiation was observed between the populations from Indian Ocean and Indo-Malay Archipelago. Hierarchical molecular variance analysis (AMOVA), pairwise F_ST_ comparisons and SAMOVA showed existence of two distinct genetic stocks of *D. russelli* in the Indian Ocean and IMA. The observed interpopulation genetic variation was high. A plausible explanation for the genetic differentiation observed between the Indo-Malay Archipelago and the Indian Ocean regions suggest the influence of historic isolation, ocean surface currents and biotic and abiotic features of the ocean. Also, there was a significant relationship between genetic distance and geographical distance between population pairs in a manner consistent with isolation-by-distance. These resulted in the evolution of a phylogeographic break for this species between these regions. The findings of these results suggest that *D. russelli* from the Indian Ocean shall be managed in its entire area of distribution as a single stock. Further, the Indian Ocean and Indo-Malayan stocks can be managed separately.

## Introduction

Understanding the population structure of any fish is of paramount importance as conservation strategies focus solely on whether the population under consideration is a single or discrete evolutionary unit. Unraveling the underlying mechanisms that lead to intra- and interspecific diversity^1^, as well as historical and recent demographic processes that collectively shape the spatial pattern of genetic diversity is key to creating conservation planning^2^. The interplay between organisms and their environment in response to ecological and anthropogenic pressures^3^ viz., overexploitation^4–6^, habitat loss and fragmentation^7–9^, climate change^10,11^, biotic exchange^12,13^, etc. have counterintuitive impact on the current evolutionary process, making it difficult to decipher and quantify their potential roles. In recent years, much research has focused on the genetic patterning and environmental adaptation of marine fish, finding populations that are genetically diverse^14^, spatially structured^15,16^ and locally adapted^17,18^. For spatially structured populations characterized by high levels of site fidelity, it is unlikely that the effects of overexploitation or habitat fragmentation at one site would have deleterious effects on neighboring populations^17^. On the contrary, if the populations are migratory and are highly interbreeding, one could easily manifest the effects on one population in nearby areas^19^.

Many marine organisms are characterized by a large effective population size and high dispersal capacity, both as larvae and adults^20^. These traits could result in high gene flow between populations over time and space unless separated by geographic or hydrological barriers that reduce migration between areas. Interspecific differences in population biology are exhibited by several migratory species. Several studies spanning a wide range of taxa from the Indian Ocean have identified many species with high levels of genetic structure^14,21–24^. However, some other species interbreed and spread extensively, resulting in low population structure^25–29^. In *Alosa pseudoharengus*, variations in patterns of genetic structure between landlocked and anadromous populations have been observed^30^. Several species of migratory fish are reported to have experienced staggering population size declines worldwide^31–33^. Pelagic sharks (*Alopias* spp., *Carcharodon carcharias* and *Sphyrna* spp.) have declined by over 75% in the Northwest Atlantic since 1986^34^. Sporadic reports of similar declines have also been recorded for other predatory marine fishes^35–37^. Failure to halt this decline will ultimately result in the loss of these species from their current habitats and has evolutionary consequences. In addition, small populations often experience high levels of genetic drift, fixation of harmful alleles and loss of genetic diversity, eventually leading to loss of genetic integrity and negatively impacting their adaptive potential^38^. Therefore, a better understanding of a species' population structure and movement patterns is required for effective conservation and equitable management of fishery resources.

The objective of the present study was to investigate the population structure of a highly mobile pelagic fish, *Decapterus russelli*. It is widespread in the Indo-Pacific from the Red Sea and East Africa to Japan and Australia^39,40^. The Indian scad, *D. russelli* represents an important fishery resource in India and Southeast Asia^40^. It represents an important pelagic resource of India, ranking next to the Indian oil sardine^41^, is an inexpensive source of animal protein^42^ and is widely used as a live fish bait^39^. Indian scad mackerel inhabits at depths of 40–275 m and are most commonly found on the coast and open shores of the Indian Ocean^39^. The species has a prolonged spawning season, with different studies reporting different spawning period overlapping along different coasts of India: February–November^43^, November–December^44^, March–December with peak in February and March^45^, December and September^46^, December–March and June–October with a peak in January and September^47^ on the southwest coast; December and August on the north-west coast^46^; April–August^46^ and December–July, sometimes extending to August^48^, November–May^49^ on the east coast of India, suggesting that this species is a continuous breeder preferring to spawn only when conditions are favorable, which can vary from place to place. Ovarian development is asynchronous and the eggs are pelagic and spherical in shape with size ranging from 0.02 to 0.97 mm^50^. The exact spawning grounds have not yet been located. The species plays an important role in the proper functioning of the ecosystem, as it avoids direct competition by increasing the number and diversity of prey items as they grow. Planktonic crustaceans (*Acetes* spp., copepods and other crustaceans) formed the main diet in smaller groups^45^ and were replaced by fish (*Lactarius* spp., *Myctophid* spp.) and molluscs^49,51,52^ in larger groups. As a primary carnivore, they inevitably help keep the ecosystem in balance as they are a voracious feeder of planktonic crustaceans^45^ and keep their population at optimal levels. They also play a crucial role in maintaining trophic ecology and the integrity of the food web. The large-scale consumption of high-energy planktonic crustaceans^45,51^ makes this highly vagile species a potential link in the food chain to temporally and spatially transfer energy to subsequent trophic levels.

*D. russelli* has been characterized primarily for its biology and population dynamics^44,48,49^, length–weight relationship^53^, diet and feeding habits^51^, reproductive biology^54–56^, and population status^46,57^. Extensive studies of its geographic structure have also been carried out from the Indo-Malay Archipelago (IMA). Previous studies have reported that two populations of scad mackerel enter the Java Sea seasonally, one during the east monsoon from the Flores Sea and Makassar Straits and another during the west monsoon from the Indian Ocean and Sunda Straits^58^. In addition, it also suggested the possibility that a third population could enter the Java Sea from the South China Sea during the west monsoon. The migration and distribution of Indian scad mackerel can be ascribed to the variation in salinity associated with the monsoon cycle in the IMA^58^. Two putative lineages have been distinguished from IMA in *D. russelli* based on the sequence polymorphism of the mitochondrial cytochrome b (*Cyt b*) gene. The two lineages reportedly showed spatial heterogeneity in their distribution. Later, Rohfritsch and Borsa^40^ discovered three mitochondrial lineages of *D. russelli* from IMA based on the combined result of mitochondrial and nuclear markers. One of the Perrin and Borsa^59^ haplogroups appeared to consist of two separate lineages in Rohfritsch and Borsa^40^. They could also observe the co-occurrence of some mitochondrial lineages, indicating recent secondary contact between once isolated populations or admixture in samples of cryptic species^60^.

Genetic information on this species from the Indian Ocean is scarce apart from a study of its complete mitochondrial genome^42^. Furthermore, with the exception of one study using truss morphometric analysis^24^, *D. russelli* from the Indian Ocean are less studied (particularly using molecular markers) than their counterparts in the Indo-Malay Archipelago. Sen et al.^24^ used 23 variables to distinguish between populations and identified separate stocks on the east and west coasts of India. The genetic basis for this observed phenotypic discreteness needs to be further elucidated in order to derive effective management recommendations for the species in different geographic areas in the Indian Ocean. Therefore, we examined the genetic population structure of Indian scad mackerel collected from five locations using mitochondrial and nuclear markers. In addition, to detect any genetic breaks between the populations of *D. russelli*, we performed a comparative study by expanding the geographic area to IMA. Since the species has high economic value and is widely harvested, the genetic data of such a species will provide adequate information to implement effective management strategies for the sustainable use of fishery resources.

## Results

### Genetic diversity analysis

#### Mitochondrial DNA (*Cyt b*) analysis

The *Cyt b* gene sequences of a total of 125 individuals sampled from five locations along the Indian coast were sequenced. After alignment and editing of ambiguous sequences, a final sequence length of 933 bp was obtained. The aligned dataset revealed an overall of 58/933 (6.21%) variable sites where 14/993 sites (1.50%) were parsimony-informative with 38 haplotypes. All haplotypes were deposited in GenBank under accession numbers OR188721 to OR188758. Furthermore, 34 (89.47%) haplotypes were singletons and 4 (10.52%) were shared between populations. The average base composition of the sequences was 23.2% A, 27.5% T, 15.4% G and 33.9% C with a higher A/T content compared to G/C. The transition to transversion ratio was 3:1. A detailed evaluation revealed 34 nonsynonymous mutations with 33 amino acid substitutions. The overall haplotype and nucleotide diversity was 0.6723 and 0.00167, respectively. The descriptive statistics of each population from the Indian Ocean are presented in Supplementary Table S1.

The previously reported IMA sequences were also included in the study^40^ bringing the total number of sequences to 583. We trimmed our sequences to match those retrieved from GenBank resulting in a final length of 307 bp. These data were used for further analysis. Altogether, the dataset revealed 30 variable sites, of which 14 were parsimony informative and 16 singleton variable sites with 27 haplotypes (Table 1) with an overall haplotype diversity (h) of 0.49684 and a nucleotide diversity (π) of 0.00593. Twenty out of the 27 haplotypes were found exclusively in the IMA, while the remaining 7 were discovered from the Indian Ocean. Out of a total of 27 unique haplotypes, only 8 (30%) were found in more than one region, with the rest being region-specific. In comparison, haplotype and nucleotide diversity in the IMA population (h = 0.25029, π = 0.00364) were higher than that in the Indian Ocean population (h = 0.10916, π = 0.00078). Furthermore, haplotype and nucleotide diversity in IMA ranged from 0.00 (Arafura) to 0.48 (Makassar) and from 0.00 (Arafura) to 0.00951 (Makassar), respectively (Table 2). The haplotype and nucleotide diversity in the Indian Ocean population ranged from 0.00 (Cochin) to 0.15667 (Mangalore and Veraval) and from 0.00 (Cochin) to 0.00261 (Mangalore), respectively. The IMA populations consisted mostly of singletons (13/20–65%) (Table 1). The average base composition of the entire sequences was 21.8% A, 31.2% T, 15.6% G, and 31.3% C. The ratio of transition to transversion was 4:1. The entire dataset revealed seven non-synonymous mutations that resulted in seven amino acid changes. It has been observed that the haplotypes shared by the IMA population are not detected in the Indian Ocean population and vice versa.

**Table 1:** Distribution of haplotypes for 15 populations of *D.* *russelli* based on *Cyt* *b* gene sequence data.

Region	East coast of India		West coast of India			Indo Malay Archipelago										Total	Haplotype frequency (%) Total
Population	Chennai	Puri	Kochi	Mangalore	Veraval	Kelang	Carita	Labuan	Tambelan	Pekalongan	Kinabalu	Sandakan	Toli-Toli	Makassar	Arafura		
Haplotype 1						1										1	0.17
Haplotype 2												1				1	0.17
Haplotype 3												1				1	0.17
Haplotype 4													22	14		36	6.20
Haplotype 5										1						1	0.17
Haplotype 6							1	2								3	0.51
Haplotype 7								1								1	0.17
Haplotype 8									1		1					2	0.34
Haplotype 9							1	1	1		1					4	0.70
Haplotype 10									1							1	0.17
Haplotype 11										1						1	0.17
Haplotype 12										1						1	0.17
Haplotype 13												1				1	0.17
Haplotype 14							1									1	0.17
Haplotype 15												1				1	0.17
Haplotype 16								1								1	0.17
Haplotype 17									1							1	0.17
Haplotype 18						1	1					1				3	0.51
Haplotype 19						10	38	72	48	64	35	61	8	8	51	395	67.75
Haplotype 20								2								2	0.34
Haplotype 21	24	23	25	23	23											118	20.24
Haplotype 22				1												1	0.17
Haplotype 23				1												1	0.17
Haplotype 24					1											1	0.17
Haplotype 25	1				1											2	0.34
Haplotype 26		1														1	0.17
Haplotype 27		1														1	0.17
Total	25	25	25	25	25	12	42	79	52	67	37	66	30	22	51	583

**Table 2:** Sample size, population genetic statistics and historical demographic analysis of 15 *D.* *russelli* populations based on mitochondrial *Cyt* *b* gene sequences.

	Indian Ocean					Non-Indian Ocean									
	East Coast		West Coast			IMA									
	Chennai	Puri	Cochin	Mangalore	Veraval	Kelang	Carita	Labuan	Tambelan	Pekalongan	Kinabalu	Sandakan	Toli-Toli	Makassar	Arafura
N	25	25	25	25	25	12	42	79	52	67	37	66	30	22	51
Number of haplotypes	2	3	1	3	3	3	5	6	5	4	3	6	2	2	1
Number of polymorphic sites	1	2	0	10	2	2	8	9	6	10	4	5	6	6	0
Haplotype diversity, Hd	0.0800	0.15667	0.00	0.15667	0.15667	0.31818	0.18351	0.16975	0.14932	0.08820	0.10661	0.14658	0.440460	0.48485	0.000
Nucleotide diversity	0.00026	0.00052	0.00	0.00261	0.00052	0.00109	0.00140	0.00177	0.00087	0.00098	0.00070	0.00068	0.00791	0.000948	0.000
Tajimas D	− 1.15753	− 1.51406	0.00000	− 2.30550	− 1.51406	− 1.45138	− 2.17642	− 1.81405	− 2.01660	− 2.33128	− 1.88391	− 1.83587	1.72038	2.37829	0.00000
Fu’s Fs	− 1.06131	− 2.12796	0.00000	0.91456	− 2.12796	− 1.32484	− 2.61585	− 2.49722	− 3.82287	− 1.84208	− 1.37648	− 6.31047	7.19054	7.43462	0.00000
*Hri* index	0.71200	0.49861	0.00000	0.60492	0.49861	0.22658	0.48821	0.58186	0.60829	0.78190	0.71515	0.59213	0.68190	0.73554	0.00000

The genetic diversity or distance values were converted to percentages and most of the genetic diversity within the population was 0%, except for Toli-Toli and Makassar which have a genetic distance of 1% (Supplementary Table S2). When populations with little genetic distance were excluded, genetic diversity between populations ranged from 1 to 2%. Makassar and Toli-Toli from the IMA differed the most of all, with at least 1% genetic distance from the remaining populations. The genetic distance of 2% was mainly observed between Indian Ocean and IMA populations (Mangalore and Toli-Toli; Mangalore and Makassar; Puri and Toli-Toli). Furthermore, within the IMA, a 2% divergence was observed between the populations of Toli-Toli and Tambalen (western region of the IMA). In addition, all other Indian Ocean populations are genetically separated from the IMA by 1%. However, subtle genetic differences were observed between the Indian Ocean populations and also between the populations from the western region of the IMA. The majority of F_ST_ values observed in the IMA were significantly different (p < 0.05) (Supplementary Table S2). In addition, significant differences in F_ST_ values (p < 0.05) were also observed between all IMA and Indian Ocean populations. However, non-significant values (p > 0.05) were found between populations from the Indian Ocean.

#### Nuclear DNA (*DrAldoB1*) analysis

A total of 125 individuals (250 alleles) of *D. russelli* from the Indian Ocean were assayed for 212 bp of intron 1 of the *Aldolase b* (*DrAldoB1*) gene. 36 unique alleles were identified of which 33 alleles were exclusively from the Indian Ocean (Table 3) and the remaining three were from IMA (IMA sequences were retrieved from NCBI-GenBank). One allele was common to all five geographical locations in the Indian Ocean (Table 3). The sequences were deposited in GenBank under accession numbers OR546246 to OR546278. Overall, the haplotype diversity was 0.3786 and nucleotide diversity was 0.01. For each population, the haplotype diversity and nucleotide diversity ranged from 0.153 (Veraval) to 0.628 (Chennai) and 0.001(Veraval) to 0.022 (Chennai) respectively (Supplementary Table S3). We observed 72 polymorphic sites out of 212 bp sequence of *DrAldoB1* of which 11 sites were the sites with more than two variants. The observed intrapopulation genetic diversity varied from 0.1% (Veraval) to 1% (Puri and Mangalore). The inter population genetic distance was negligible between the Indian Ocean populations (Supplementary Table S4). Whereas, all of the Indian Ocean population displayed a higher genetic distance between IMA, ranging from 1.3% (Veraval) to 1.9% (Puri) (Supplementary Table S4. The observed global F_ST_ value was low (0.052). Pairwise F_ST_ analysis revealed a lack of genetic structure in the areas studied from the Indian Ocean and was not significant (p > 0.05) (Supplementary Table S4). However, significant F_ST_ values (p < 0.05) were obtained between IMA and Indian Ocean populations.

**Table 3:** Unique alleles of *Aldolase* *b* detected in *D.* *russelli* population from the Indian Ocean.

Alleles	Population					Total
	Chennai	Puri	Cochin	Mangalore	Veraval	
1					2	2
2					2	2
3	30	46	39	36	46	197
4		1				1
5		1				1
6		1				1
7		1				1
8				2		2
9				2		2
10				1		1
11				1		1
12				2		2
13				2		2
14				2		2
15				2		2
16	6					6
17	1					1
18	1					1
19	3					3
20	1					1
21	2					2
22	2					2
23	1					1
24	1					1
25	1					1
26	1					1
27			1			1
28			1			1
29			2			2
30			2			2
31			3			3
32			1			1
33			1			1
Total	50	50	50	50	50	250

### Population structure

#### Within Indian Ocean analysis

AMOVA analysis was performed on the 5 populations from the Indian Ocean subdivided into 1-4 gene pools. In all analyses, the overall levels of genetic differentiation between coasts were non-significant (p > 0.05) even for the highest variance (F_CT_ = 0.01090, p > 0.05) with a global F_ST_ of 0.01030. The genetic differentiation among population within groups/coast was − 0.06% (F_SC_ = − 0.00061, p > 0.05). 98.97% percentage of the total variation of *D. russelli* from the Indian Ocean is contributed by the genetic differences among the total populations. Furthermore, AMOVA analysis undertaken with nuclear markers also demonstrated the lack of significant genetic structure (p > 0.05) in *D. russelli* (Supplementary Table S5). This result indicated no subdivision or structuring within the tested populations. Additional genetic differentiation analysis revealed that estimates based on H_ST_ (0.006), N_ST_ (0.002) and K_ST_^*^ (0.001) were all very low and none of them were significant (p > 0.05). This also indicates a high level of gene flow among the sampled *D. russelli* populations from Indian Ocean (Nm = 207.34 for haplotype-based statistics and Nm = 83.97 for sequenced based statistics). On the other hand, the IMA population, divided into two geographical groups, revealed significant genetic structuring at all levels (Table 4). The mantel test showed no significant correlation between genetic distance (pairwise F_ST_ values) and geographical distance (r = 0.063, p > 0.05) among the tested population (Supplementary Fig. S1). Overall, 4 and 3 populations out of 5 were significant with negative values of Tajimas’ D and Fu’s Fs (Supplementary Table S1) suggesting historical population expansion at these sites associated with an excess of recent mutations or rare alleles^61^. The Harpending raggedness index (*Hri* = 0.46, SSD = 0.03, P = 0.02) corroborated these results. Mismatch analysis revealed a unimodal distribution providing strong evidence for a sudden population expansion (Supplementary Fig. S2). The minimum spanning network (MSN) produced a reticulation of 38 haplotypes (Supplementary Fig. S3). One haplotype was common to all geographical locations. The common haplotypes within the network are inferred to be ancestral, whereas the tip haplotypes may be derived or descended from ancestral (internal) haplotypes^62^. Thus, the occurrence of star like pattern radiating from these major haplotypes suggests that the *D. russelli* population has undergone a recent demographic expansion. Phylogenetic analysis based on mitochondrial and nuclear markers revealed no obvious phylogeographic pattern separating the 5 samples of *D. russelli* (Supplementary Fig. S4; Supplementary Fig. S5).

**Table 4:** Results of the analysis of molecular variance (AMOVA) for *D.* *russelli* showing F-statistics analysis of mitochondrial *Cyt* *b.*

Hierarchical level	Variation (%)	F-statistic	P value
Among IO			
Among regions (F_CT_)	1.09	0.01090	ns
Among populations within region (F_SC_)	− 0.06	− 0.00061	ns
Among individuals within populations (F_ST_)	98.97	0.01030	ns
Among IMA			
Among regions (F_CT_)	83.29	0.83288	*
Among populations within region (F_SC_)	− 0.08	− 0.00464	*
Among individuals within populations (F_ST_)	16.79	0.83211	*
Between IO and IMA			
Among regions (F_CT_)	72.09	0.72094	*
Among populations within region (F_SC_)	14.14	0.50668	*
Among individuals within populations (F_ST_)	13.77	0.86233	*

*IMA* Indo-Malay Archipelago, *IO* Indian Ocean.

*ns* not significant (P > 0.05).

*P < 0.01.

#### Between Indian Ocean and IMA analysis

The AMOVA analysis was also performed on the 15 populations (10 from the IMA and 5 from the Indian Ocean) that were subdivided into two geographical groups (Group 1: Indian Ocean; Group 2: IMA) to determine whether there is any genetic structuring over the geographical area. AMOVA analysis revealed that genetic differentiation was significant at all levels (Table 4), with 72.09% of the total variation attributed to variation among regions/groups, implying significant differentiation across geographical areas. The analysis was also repeated with the IMA populations divided into three geographical locations. The test is to discover whether the genetic differentiation was visible at the finer spatial scales across the IMA. Thus, the sample was divided into 4 groups (Group 1: Indian Ocean; Group 2: Makassar strait/Sulawesi Sea; Group 3: Arafura Sea; and Group 4: western region of the Indo-Malay Archipelago). The analysis generated a non-significant albeit low F_SC_ value, whereas significant differentiation among groups (F_CT_ = 0.8267, p < 0.05) was observed accounting for the highest percentage (82.77) of the total variation. The analysis was repeated splitting the Indian Ocean population into two groups based on coast, bringing the total group to five. The groups were, Group 1: Western Indian Ocean; Group 2: Eastern Indian Ocean; Group 3: Makassar strait/Sulawesi Sea; Group 4: Arafura Sea; Group 5: western region of the IMA. It produced similar outcomes as the previous group of four. In addition, genetic differentiation estimates were analyzed by dividing the whole population into two: Indian Ocean and IMA. All values generated (H_ST_ = 0.60, N_ST_ = 0.79 and K_ST_^*^ = 0.54) were high and significant. *DrAldoB1* also revealed significant genetic structuring (p < 0.05) between IMA and Indian Ocean populations (Supplementary Table S5).

The result from the SAMOVA was consistent with other analyses, with k = 2 showing the highest F_CT_ value (F_CT_ = 0.8362, p < 0.05), clearly demonstrating the existence of two genetically distinct *D. russelli* stocks among the populations under study, namely the IMA and Indian Ocean populations. The mantel test showed a significant correlation between genetic differentiation (pairwise F_ST_) and geographical distance (r = 0.11, p < 0.05) among the tested population.

### Phylogenetic relationship

**Figure 1:**
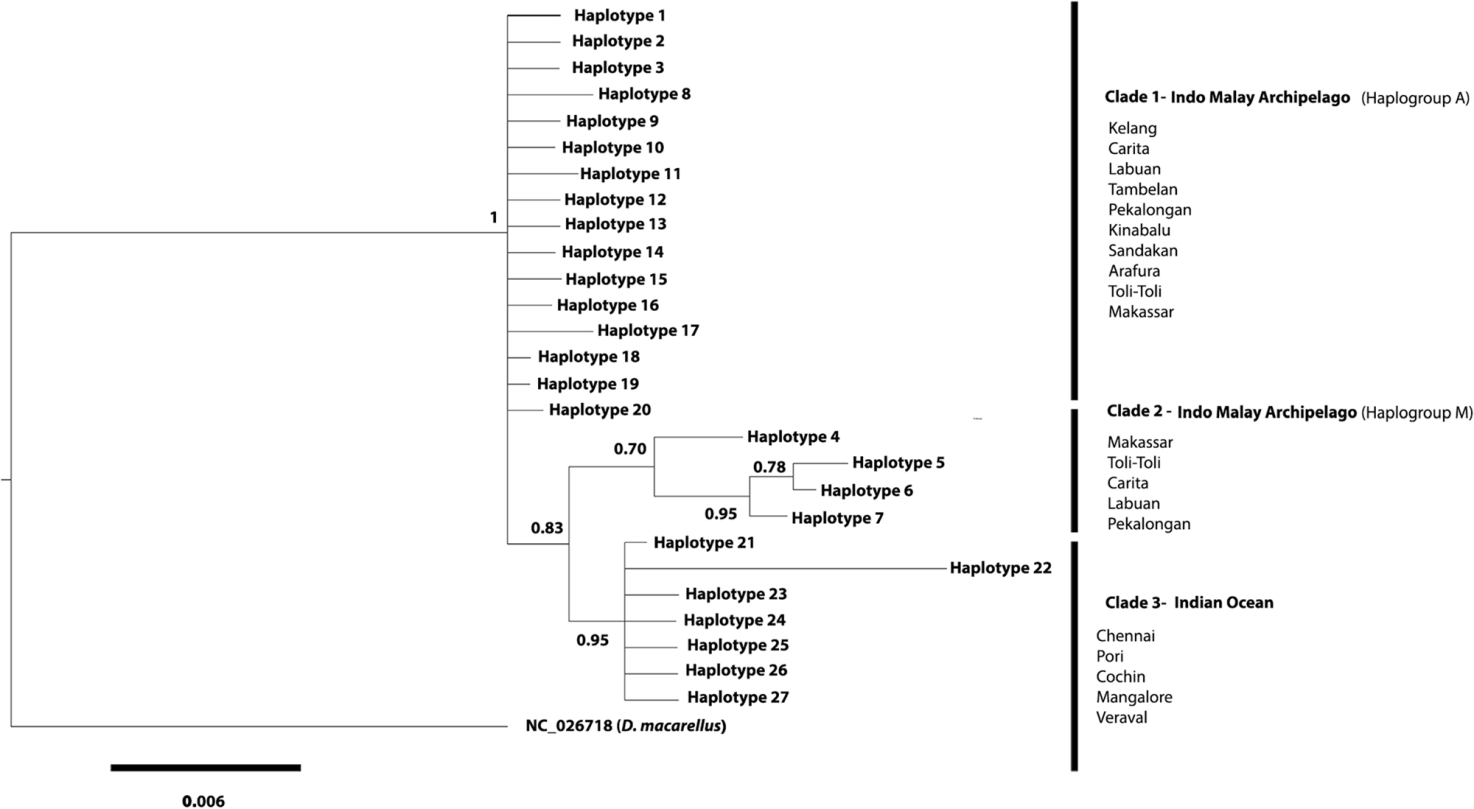
Bayesian inference phylogenetic relationships among 27 mtDNA *Cyt b* haplotypes in *D.* *russelli.* Bayesian posterior probabilities are superimposed with each node. The phylogenetic tree was generated using MrBayes 3.2.7.

The Bayesian phylogenetic tree revealed three mitochondrial lineages (Fig. 1). The two lineages were haplogroups M and A formerly identified by Perrin and Borsa^59^. Two distinct mitochondrial lineages with heterogeneous geographic distribution were found for Toli-Toli, Pekalogan, and Labuan. The third lineage consisted of populations from the Indian Ocean. In addition, the Perrin and Borsa’s^59^ M haplogroups formed a sister lineage with the populations from the Indian Ocean. The two major lineages were observed to be separated by an average nucleotide divergence of 2%. Haplogroup M and the Indian Ocean population were separated from haplogroup A by an average nucleotide difference of 2.2% and 1.8% respectively, when the three lineages were considered. In addition, the haplogroup M and the Indian Ocean population are separated by a genetic divergence of 2.2.%.

**Figure 2:**
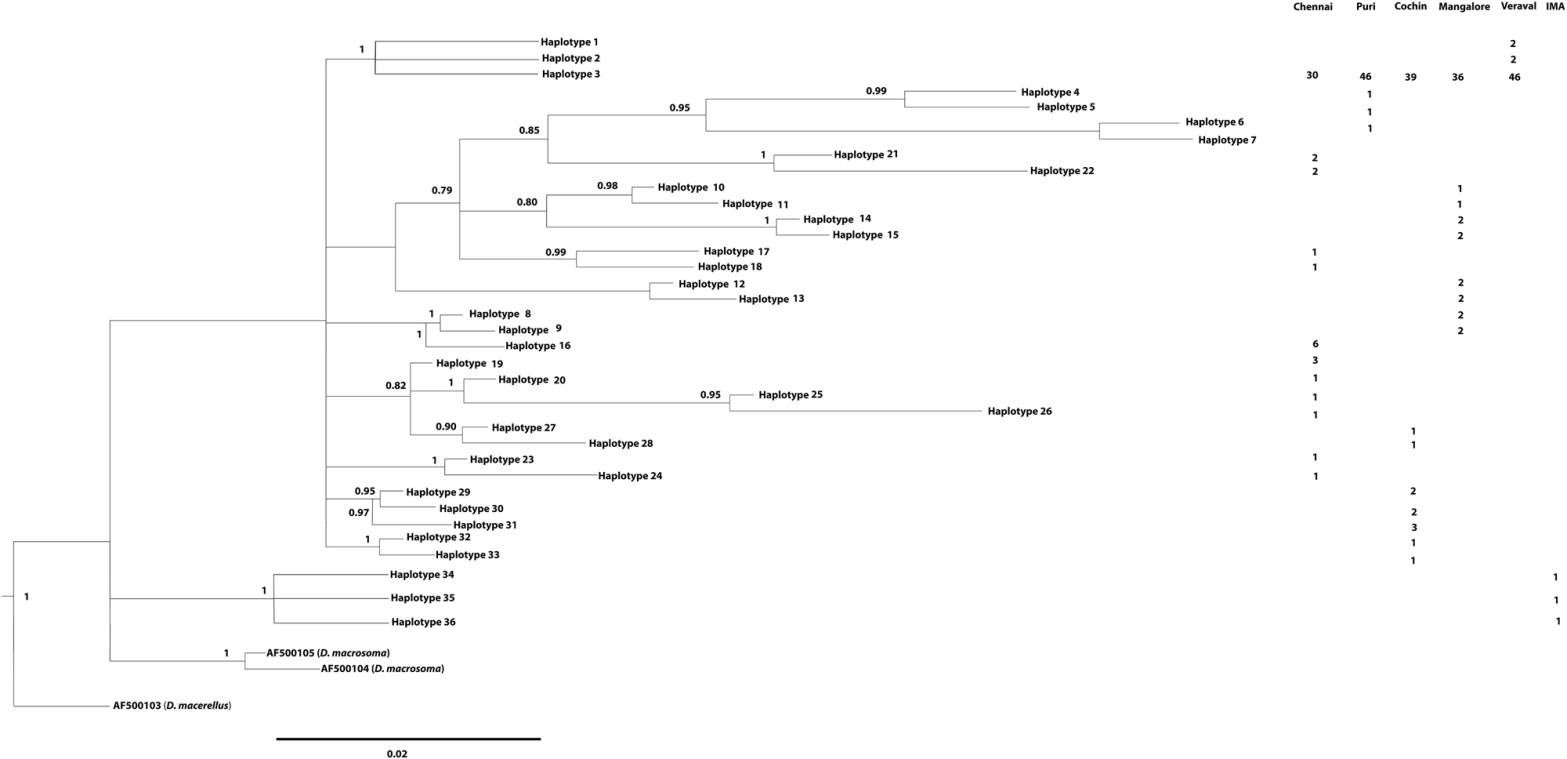
Bayesian Inference phylogenetic relationships among 36 alleles of Intron 1 partial sequences of *Aldolase* *b* gene of *D.* *russelli*. Bayesian posterior probabilities are superimposed with each node. The phylogenetic tree was generated using MrBayes 3.2.7.

The Bayesian phylogenetic tree produced from *DrAldoB1* formed 2 lineages representing an Indian Ocean lineage and an IMA lineage (Fig. 2). These two lineages were observed to be separated by an average nucleotide divergence of 1.6%.

### Haplotype network diagram

**Figure 3:**
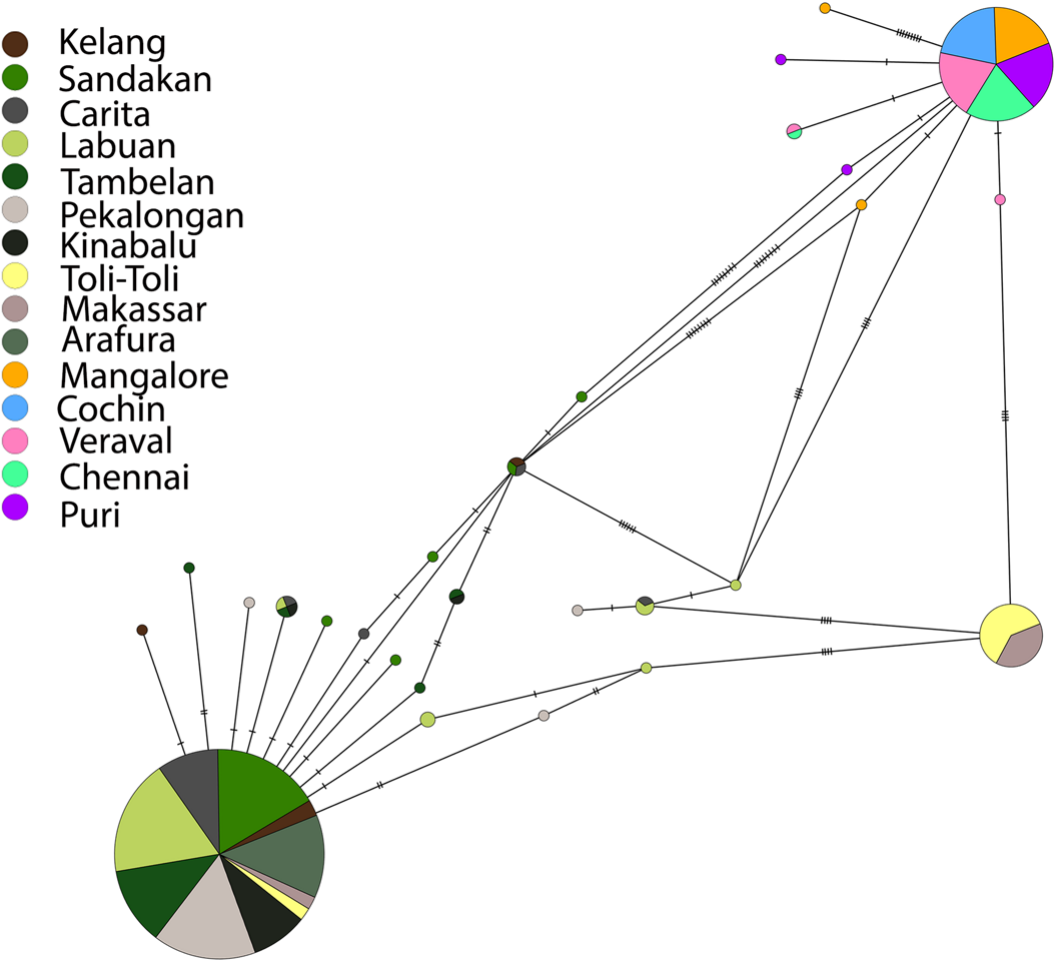
Minimum spanning network inferred from mitochondrial *Cyt* *b.* Colored circles represent different regions (refer to legend). The network diagram of all haplotypes was constructed based on median joining calculations in PopARTv1.7.

The haplotype network diagram (Fig. 3) derived from partial sequences of the mitochondrial *Cyt b* gene, delineated three major clusters similar to the phylogenetic tree: haplogroup A, haplogroup M and Indian Ocean populations. The rarer haplotypes from IMA, *h1*, *h2* and *i* comprising the M haplogroup formed a distinct cluster separated from haplotype *M*, the most frequent haplotype in the haplogroup M by 4 genetic mutations, supporting the finding of Perrin and Borsa^59^. The haplogroup M has been separated from haplogroup A by 6 mutational steps. The cluster formed by the Indian Ocean population was separated from haplogroups A and M by 8 and 4 mutational steps, respectively.

### Demographic history

Overall, the 11 and 10 populations out of the total populations tested were significant with negative Tajimas D and Fu’s Fs (Table 2) values respectively, indicating a recent historical directional selection (selective sweep) or population expansion^63^ at these sites. In addition, the Harpending Raggedness index, *Hri* = 0.5, SSD = 0.04 (p = 0.2), corroborated this finding with the recent population growth hypothesis^64^. Furthermore, based on the mismatch distribution of the combined population, past population demography of *D. russelli* revealed three highly divergent peaks, representing the three lineages (Supplementary Fig. S2). A bimodal mismatch distribution was found in the IMA lineage 2 after additional independent analysis of the three lineages. However, further comprehensive studies from the IMA are needed to confirm this preliminary finding. The IMA lineage 1 and the Indian Ocean (IO) lineage on the other hand, has a unimodal distribution indicating a rapid expansion of its population.

The time of demographic and spatial expansion for the major lineages 1 (IMA lineage 1) and 2 (IMA lineage 2 + IO lineage) using the formula τ = 2 μt are estimated to occur 8,58,934–4,46,254 years ago and 4,57,680–4,46,254 years ago respectively. Therefore, the spatial expansion coincided with the demographic expansion. Furthermore, the spatial and demographic expansion of IO lineage occurred between 6,11,285–3,17,589 years ago. In addition, the demographic expansion of lineage 2 (IMA lineage 2) occurred between 7,93,1596–3,17,589 years ago, whereas the spatial expansion of its population was between 3,66,771–1,90,553 years ago. Therefore, this lineage experienced demographic expansion following spatial expansion.

## Discussion

Genetic data based on mitochondrial and nuclear markers revealed little divergence among the five populations analyzed, suggesting the existence of panmictic populations of Indian Scad, *D. russelli* from Indian waters. The lack of significant genetic differentiation between populations as indicated by AMOVA analysis (F_ST_ > 0.05) suggests that *D. russelli* populations can be managed as a single stock in Indian waters. These findings contradicted the previous study which suggested the presence of two distinct stocks of *D. russelli* along the east and west coasts of India^24^. The previous study was conducted based on truss morphometry over a small geographical area, with opportunistically collected individuals over a short period of time probably resulting in a potential artifact of population subdivision. Morphometric and meristic characters are particularly vulnerable to environmental influences and are considerably impacted once the species is exposed to environmental stressors. These environmental stressors act as selection pressures affecting the structure and function of organisms, reducing their Neo-Darwinian fitness^65^. Environmental conditions such as currents or water masses and various stressors, including biotic and abiotic factors such as food availability, salinity and temperature are subject to temporal and spatial variability and affect the morphometry of the species at each site at any given time. Although it is widely acknowledged that the environment plays a role in shaping the phenotype, there are additional important factors influencing phenotypic variation that are being investigated at the molecular level^66^. Thus, the variations reported by Sen et al.^24^ in the specimens from the Arabian Sea and Bay of Bengal can be proposed as a consequence of phenotypic plasticity in response to temporal variations in environmental and oceanographic parameters.

In this study, the high haplotype diversity, Hd (values ranging from 0.43 to 0.90) coupled with low nucleotide diversity, π (ranging from 0.0007 to 0.002) observed in all populations was concordant with previous reports of several pelagic marine species from the Indian Ocean. Previous studies from the Indian Ocean include *Euthynnus affinis* (Hd = 0.9, π = 0.01)^67^, *Xiphias gladius* (Hd = 0.88 π = 0.002)^68^, *Rastrelliger kanagurta* (Hd = 0.95 π = 0.008)^69^ and *Thunnus obesus* (Hd = 0.99 π = 0.04)^27^. High haplotypic diversity within populations can be maintained for a variety of reasons, including large population sizes, environmental variation, and life-history factors that favor rapid population growth^70,71^. The high-level haplotype diversity observed in this study may be due to the large effective population size and wide distribution as suggested for other pelagic fishes^72,73^. *D. russelli* is also the most abundant carangid in Indian waters^41^.

It has been proposed that population history of marine species can be classified into four categories based on different values of haplotype diversity (Hd) and nucleotide diversity (π) of mt DNA gene sequences^74^. High Hd values and low π values observed in the present study indicate a recent population expansion from a small effective population size, i.e. a rapid population increase is seen in *D. russelli* population after a bottleneck event with mutation accumulation (second category; Grant and Bowen^74^). The unimodal mismatch analysis, the significant negative Fu's Fs and Tajima’s D values, non-significant deviation from the sum of squared deviations (SSD) and the Harpending’s raggedness index (*Hri*) were also consistent in supporting the occurrence of population expansion of *D. russelli* in Indian waters.

Marine fishes are reported to have strong intraspecific gene flow than freshwater fishes and hence reduced genetic structure^75^. This is probably because the marine environment imposes fewer barriers to species dispersal. In particular, there are reports of low levels of genetic differentiation in species with a pelagic life history both within^27,67–69^ and between the oceans^25,68,76^, largely due to the existence of a continuous circumglobal pelagic environment and the presence of a wide range of suitable spawning grounds. One of the possible explanations for the observed lack of population subdivision in *D. russelli* samples includes seasonal fluctuations in water circulation caused by the monsoon currents in the Indian Ocean. During the northeast monsoon, the flow of the upper ocean is directed westward towards the Arabian sea. The flow direction changes during the southwest monsoon, flowing eastward from Somalia towards the Bay of Bengal^77^. This seasonal reversal of monsoon currents may homogenize *D. russelli* populations in Indian waters. In addition, the Indian Ocean is divided into two hemispheres by the equator, resulting in the well-known marine bioecological impacts determined by latitudes^68^. Furthermore, this ocean is characterized by the westward South Equatorial Current (SEC)^78^ and hydrochemical south Tropical Front which separate the two large oligotrophic areas—Indian Monsoon Gyre Province (MONS) in the north and the Indian South Subtropical Gyre Province (ISSG)^79^. However, these physical and ecological divisions have little impact on the genetic structure of *D. russelli* in contrast to the Indo Malay Archipelago counterparts. In addition, the life history traits of *D. russelli* like long distance migration^58^, fast growth rate^53^ and high fecundity^55^ contribute to a high level of gene flow, which in turn led to the low level of genetic subdivision in this species. Furthermore, the phylogenetic groups that were evident from the Bayesian analysis of both *Cyt b* and *DrAldoB1* genes data of *D. russelli* revealed the absence of geographical clustering of lineages among the Indian Ocean populations. Moreover, the haplotypes from all samples were interspersed throughout the tree.

Analysis of mitochondrial *Cyt b* and *DrAldoB1* sequences indicated strong isolation between the Indian Ocean and IMA with significant level of genetic differentiation. The Indian Ocean is impacted by climatic variability on a larger scale^77^. It displays a variety of climate variability modalities, most of which are associated with the robust seasonal cycle ranging from intra-seasonal to inter-annual and even longer time scales. It differs from the other oceans in a number of climatically important ways^78^—first, the Asian landmass blocks the northern entrance to the Indian Ocean, preventing heat export to the north. Second, the strongest monsoon on earth originates from the Asian continent. These monsoonal winds cause significant seasonal variations in ocean currents including annual reversals. In addition, unlike other oceans, the Indian Ocean does not experience consistent equatorial easterlies, which prevents it from experiencing climatological equatorial upwelling. Finally, the Indian Ocean has the Indonesian Throughflow (ITF), a low-latitude exchange route with the Pacific. The central Indo-West Pacific region is geographically and hydrologically complex and possesses the world’s largest marine diversity hotspot^80^. The region experienced major changes to its shorelines during the Pleistocene^81,82^. The shallow seas of the Sunda shelf and Sahul shelf (Arafura Sea) were above the current sea level during the Pleistocene. Land barriers are also being built to separate the South China Sea from the Indian Ocean at the southernmost point and from the Sulu Sea to the east. The repeated fluctuation in sea level associated with the ice ages may be the reason for the geographic isolation of several inland seas, leading to the genetic differentiation of the species they harbored. This is believed to be the reason for the geographic discontinuity observed in many marine species between the Indian and Pacific Oceans and also for the greater species diversity observed in the Indo-Malay region^81^. These environmental factors together with the emergence of land barriers to movement between ocean basins appear to be the strongest factor for the observed genetic heterogeneity of *D. russelli* populations between the Indian Ocean and the IMA. This may also be responsible for shaping the modern genetic structure of many species that inhabit this region as we see it today^81–85^.

Three phylogenetic groups were evident in the *Cyt b* data for *D. russelli*. The tree showed evidence for the existence of distinct geographical lineages present among IMA and between IMA and Indian Ocean populations. All IMA sites grouped in Clades 1 and 2 correspond to the haplotypes A and M recognized by Perrin and Borsa^59^ and Rohfritsch and Borsa^40^ with an obvious pattern of geographic structuring. In clade 2, one lineage is consists entirely of Makassar and Toli-Toli individuals (haplotype M) and the next lineage encompasses haplotypes *h1, h2 and i* as in Perrin and Borsa^59^ and Rohfritsch and Borsa^40^. Clade 3 consisted entirely of Indian Ocean individuals. The previous reports of the co-occurrence of separate mitochondrial lineages in some samples and their heterogeneous geographical distribution (western populations and easternmost populations)^40,59^ were confirmed in this study. Due to the high connectivity of the seas in the Indo-Malay Archipelago, one might anticipate a high level of gene flow between populations. However, seasonal variations in the surface currents in the western part of the archipelago in response to the typical monsoon cycle may cause changes in water circulation and salinity, which can affect the migration patterns of adult pelagic species and the dispersal of their larvae^58^. However, the divergence of these clades may have occurred in the past during the Pleistocene lowering of sea level.

The average rate of nucleotide changes in the *Cyt b* gene observed in this study (1.8–2.2%) between various lineages lies in the range described for marine fishes (between the extremes of sharks and mammals (2.5% and 1.0% per million years respectively)). This indicates that the split between the lineages dates back to the mid-Pleistocene. In addition, the co-occurrence of the Haplogroup M of Rohfritsch and Borsa^40^ and the Indian Ocean population to form a major lineage and their heterogeneous geographical distribution suggests that differences may be due to vicariance. The average genetic divergence between the haplogroup M and the Indian Ocean population corroborates the above findings (while considering the whole population (2.2%) vs only these two (haplogroup M and IO population) (1.8%)).

Past geological and climatic events have undoubtedly played a major role in this inferred population expansion. The paleogeography of IMA changed dramatically during the Quaternary period^10^. During the last interglacial period, there were marine connections between the Indian Ocean and the south China Sea via the Strait of Singapore^85^. These connections may have allowed *D. russelli*, an essentially pelagic species to freely migrate and expand its population across this region at that time. Estimation of the time since expansion of various lineages of *D. russelli* suggests the timing of these events is consistent with the cyclic sea level rises that occurred during the late Pleistocene between 1,600,000 to 10,000 years ago^86^. The land bridges, which were later exposed as sea level fell, impeded the spread of *D. russelli*. Furthermore, the last glacial maximum (LGM), 30,000 to 19,000 years ago led to the final decline in sea level up to the present^86,87^. A substantial portion of Sunda and Sahul shelves was exposed as the sea level declined with glaciation up to 200 m below their present level^82^. This process would have also impeded the dispersal of many marine taxa. By this time the adjacent South China Sea considerably shrank in size and transitioned into a semi closed marginal sea exposing a massive low gradient on the Sundaland carton^86^. In addition, around this time the modern Malayan Penisula, Borneo and Sumatra formed highlands to the south^88^ adding yet another potential barrier to population migration. The co-occurrence of an IMA lineage and an IO lineage (clade 2 and clade 3) as well as their distinct geographic distributions, as seen in this study, can be attributed to the remnants of these earlier marine links and the emergence of geographic barriers brought about by ice ages associated with the Pleistocene events respectively. Such isolated marine populations experienced extensive inbreeding leading to the accumulation of genetic variation and the structuring of their population at the genetic level. Similar scenarios have been reported for the genetic heterogeneity of many marine species between oceans^68,69^. MSN analysis also supported this structuring of *D. russelli* across the IMA and between the Indian Ocean and the IMA.

Marine species with high dispersal potential usually display high level of gene flow resulting in low level of genetic structuring^89^. This would limit or reduce adaptive divergence among local populations^90^. The low and non-significant F_ST_ values observed here among the five *D. russelli* populations from Indian waters are consistent with the pattern of *Cyt b* homogeneity and high gene flow (Nm = 208.34). Various evolutionary processes including migration, mutation and drift may play a role in determining genetic differentiation between populations. Thus, a highly migratory species with a large population has a tendency to show low level of population differentiation in contrast to species with small populations and reduced migration rates^91^. Several other investigations have also reported relative panmixia in marine species across Indian waters. For example, an apparent lack of genetic structure was observed in Bigeye tuna (*Thunnus obesus*)^27^ and *Euthynnus affinis*^67^ based on the analysis of mitochondrial control region. Furthermore, mitochondrial (*ND2*) and nuclear DNA (microsatellites) analyzes of *Xiphias gladius* have shown its homogenous distribution throughout the Indian Ocean^68^.

The present study also identified a significant population differentiation between the IMA and the Indian Ocean (Nm = 0.13). Although it is true that gene flow between populations can often prevent subpopulation isolation^92,93^, this relationship is not as simple as it first appears. The hierarchical AMOVA results revealed significant F_ST_ and F_CT_ values indicative of the genetic structure among populations of *D. russelli*. This was confirmed by spatial genetic heterogeneity (k = 2 in SAMOVA) between groups comprising the populations in the IMA vs Indian Ocean. In addition, the geographical distance also contributed to the divergence among populations. Overall genetic distance increased with geographical distance consistent with isolation by distance. Thus, a more plausible explanation for the observed population differentiation of two distinct groups of *D. russelli* is the combined effects of several factors including historical physical isolation during the Pleistocene, larval dispersal factors, biotic and abiotic features of the ocean basins, ocean currents and geographical distance.

The movement of marine fishes, especially as larvae and adults, can greatly be influenced by Ocean surface currents. In the Indian Ocean, it is largely influenced by monsoon currents, also known as monsoon drifts, which flow between the Bay of Bengal and the Arabian Sea^94^. In contrast, the Kuroshio current, the largest surface current in the South China Sea affects the waters surrounding the IMA^95^. These two sea surface currents can contribute to, and also act as, powerful factors in genetic differentiation among *D. russelli* populations between the IMA and the Indian Ocean.

The significant genetic differentiation detected between Indian Ocean and IMA needs to be corroborated by advanced genomic markers like SNPs detected through genome scans. Genome scans also will provide insights regarding loci under selection in response to heterogeneous environmental conditions of the Indian ocean and IMA^96,97^. In addition, tagging programs and microchemical analyzes of specific structures (eg: otolith) may also be helpful to devise management measures for *D. russelli* in the Indian Ocean. Finally, a comprehensive understanding of reproductive strategies, population dynamics in various regions, and spawning areas within the Indian Ocean is required to fully understand the nature and behavior of its population^98^.

## Conclusion

The results of this study demonstrated that the Indian Ocean *D. russelli* constituted a single panmictic population and can be managed as a single stock. This study revealed two discrete populations of *D. russelli*, Indian Ocean populations and IMA populations. The inferred demographic history indicates that *D. russelli* populations may have experienced a potential population expansion during the Pleistocene. The subsequent appearance of a barrier to gene flow may have limited their ability to migrate between the Indian Ocean and the IMA as seen in the extant populations.

## Materials and methods

### Sample collection

**Figure 4:**
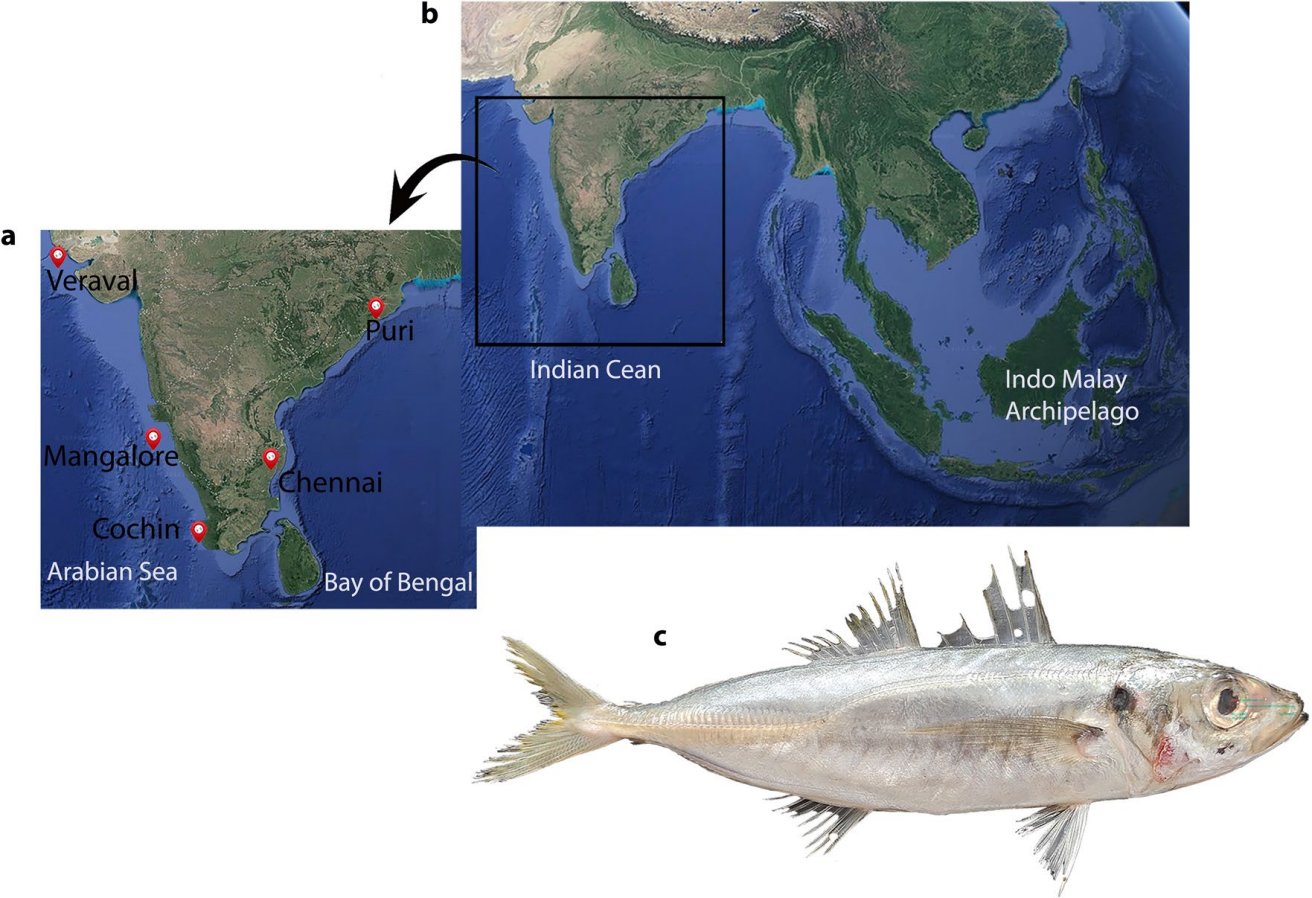
Map of sampling sites, site used for comparative analysis and targeted species. (**a**) Samples were collected from respective landing sites (red pin) from 5 localities of the Indian Ocean. (**b**) A comparative analysis of *D.* *russelli* populations in the Indian Ocean and the Indo-Malay Archipelago. (**c**) Specimen of Indian Scad, *D.* *russelli.* The map was obtained from Google Earth and the sampling sites were marked using the placemark tool in Google Earth available at http://earth.google.com. The images were then combined and edited manually in Adobe Photoshop CS6.0.

A total of 125 *D. russelli* individuals were collected from the east (Odisha (Puri) and Tamil Nadu (Chennai)) and west (Gujarat (Veraval), Mangalore (Karnataka) and Kerala (Cochin)) coasts of India between 2019–2020 (Fig. 4) A piece of caudal fin basal tissue was collected from each individual and preserved in 95% ethanol until DNA isolation. The fish sample used in this study was treated in accordance with the recommendations made by De Tolla et al.^99^ for the handling and use of fish in research. The protocols were approved by the ethical committee of the ICAR—Central Marine Research Institute, Kochi. These methods are also reported following ARRIVE guidelines (http://arriveguidelines.org).

### DNA isolation and PCR amplification

Genomic DNA was isolated from tissue using the standard phenol–chloroform protocol^100^. The isolated DNA was then eluted in T.E. buffer (1 ×) and the DNA samples were stored at 4 °C prior to amplification by PCR. The quality and quantity (concentration) of the extracted DNA was checked using gel electrophoresis or NanoDrop One Spectrophotometer (Thermoscientific, USA). To facilitate comparative analysis, a new primer set was designed that targets a 933 bp fragment of mitochondrial *Cyt b* gene sequences spanning the regions analyzed by Rohfritsch and Borsa^40^. The primer sequences were as follows: 5′-AATCTCCGTAAAACCCACCC-3′ (forward) and 5′-AAAAACTAGGAATAGGAGGAAGT-3′ (reverse). The primers were designed based on the aligned full-length *Cyt b* gene sequences of carangids using Geneious R7 (Biomatters Ltd., New Zealand). The PCR reaction mixture consisted of 15.6 μl of sterile deionized water, 2 μl of 10 × buffer, 10 mM of each dNTP, 10 μM of each oligonucleotide, 1 unit of Taq DNA polymerase, and ~ 50 ng of genomic DNA as template. Amplification conditions were: initial denaturation at 94 °C (4 min); 32 cycles of 94 °C (30 s), 55.4 °C (30 s), 72 °C (1 min 10 s) and a final extension at 72 °C (10 min) before the reaction termination at 4 °C. Amplification of this 20 μl reaction volume was performed in a Biorad T100 thermal cycler (Bio-Rad, USA). Intron 1 of the aldolase b (*DrAldoB1*) gene was targeted with the following primers; 5′-GCTCCAGGAAAGGGAATCCTGGC-3′ (forward) and 5′-CTCGTGGAAGAAGATGATCCCGCC-3′ (reverse)^101^. PCR amplification was performed in a Biorad T100 thermal cycler (Bio-Rad, USA) with a final volume of 25 μl, consisting of 12.5 μl of 2 × EmeraldAmp^®^ GT PCR Master Mix (TaKaRa, Shiga, Japan), 0.3 μl of each primer (10 mM each), 2.0 μl of genomic DNA (50 ng/ml) followed by nuclease-free water to achieve final reaction volume. Thermal cycling conditions were as follows: initial denaturation at 94 °C for 4 min, followed by 35 cycles of 30 s denaturation at 94 °C, 12 s annealing at 52 °C, 20 s extension at 72 °C followed by final extension of 3 min at 72 °C. DNA amplification products were then separated in 1.5% agarose gels at 90 V in 1× Tris–borate–EDTA (TBE) buffer, stained with ethidium bromide and visualized using a gel documentation system (Bio-Rad, USA). All PCR products were then sequenced using Sanger sequencing in both the forward and reverse directions. The nucleotide sequences were checked visually to ensure that the sequence information was consistent in both directions. The sequences were also verified by comparison with known sequences from GenBank.

### Data analysis

Both forward and reverse raw sequences were edited and assembled after removing the ambiguous bases in MEGA X^102^. The edited sequences of each locus were aligned with Clustal W implemented in the same software. The coding sequences of *Cyt b* were translated into amino acids, checked for stop codons and then back translated into the corresponding nucleotide sequences. A total of 933 bp sequences of *Cyt b* and 212 bp sequences of *DrAldoB1* were obtained for the subsequent analysis. Further, *Cyt b* (accession numbers: AF307494–AF307510 and AF515757–AF515759) and *DrAldoB1* (accession numbers: AF500103–AF500105) gene sequences deposited from IMA were also retrieved for comparative analysis. Prior to data analysis, the PHASE^103^ program implemented in DnaSP6^104^ was used to resolve heterogeneous sites in *DrAldoB1* sequences and to reconstruct haplotypes.

### Genetic diversity analysis

Genetic distance within and between populations was estimated using K2P model, selected based on the lowest Bayesian information criterion (BIC) score using MEGA X^102^. These values were also used to assess the possibility of occurrence of subspecies or cryptic species (if intra-specific variation exceeds the threshold of 2% in marine species)^105–108^. The complete aligned dataset was analyzed for nucleotide variable sites, parsimony informative sites, haplotype number, haplotype frequencies and nucleotide frequencies in DnaSP6^104^. The ratio of transition to transversion was analyzed using MEGAX^102^.

### Population genetic structure analysis

Population pairwise F_ST_ values were calculated using ARLEQUIN v3.5^109^. Hierarchical analysis of molecular variance (AMOVA) was executed to estimate population subdivision and structuring at different hierarchical levels using ARLEQUIN v3.5^109^. The AMOVA analysis was performed at three levels: among the Indian Ocean population (1), among the IMA population (2) and between the Indian Ocean and the IMA populations (3). For the Indian Ocean population, a hierarchical AMOVA analysis was performed by partitioning the variance among and within populations. (i) one gene pool comparison considering the whole population as a single gene pool, (ii) two gene pools [West coast (Cochin, Mangalore and Veraval); East coast (Chennai and Puri)] (iii) three gene pools [North West (Veraval); South West (Mangalore and Cochin); East (Chennai and Puri), and (iv) four gene pools [North West (Veraval); South West (Mangalore and Cochin); South East (Chennai) and North East (Puri)]. Further the variation was assessed among groups, within groups and within populations. The IMA population was divided into two geographically distinct gene pools based on the previous findings on their distribution^40^: Makassar Strait and Sulawesi Sea populations vs the populations from the entire western region of IMA. For the comparative analysis, the whole data set was divided into two groups: one comprising the populations from the Indian Ocean and the other comprising the entire population from the IMA. The variance among populations and the relative contribution of variance were estimated at three different levels; F_ST_ (variation among individuals within populations), F_SC_ (variation among populations within region) and F_CT_ (variation among regions)^110^. In addition, the spatial structure was examined using spatial analysis of molecular variance (SAMOVA) v2.0^111,112^ to identify groups of populations that were geographically homogenous and maximally differentiated from each other. Gene flow (Nm) between populations based on both haplotype and sequence statistics was estimated according to Nei^113^ and Hudson et al.^114^ using DnaSP6^104^. Genetic differentiation estimates based on H_ST_, N_ST_ and K_ST_^*^ were determined in DnaSP6^104^. The isolation by distance or Mantel test has been conducted to confirm the relationship between genetic distance and geographical distance^115^.

### Phylogenetic analysis

The phylogenetic relationship among haplotypes was examined by constructing a phylogenetic tree based on Bayesian inference (BI) in MrBayes with 1,000,000 Markov Chain Monte Carlo (MCMC) generations. Posterior distributions were sampled for every 10,000 generations. The sequence of *D. macarellus* (NCBI, GenBank Accession No. NC_026718) was employed as an outgroup for phylogenetic tree construction based on *Cyt b* sequences and the sequences of *D. macarellus* (Accession No. AF500103) and *D. macrosoma* (Accession No. AF500104, AF500105) were used as outgroups for phylogenetic tree construction based on *DrAldoB1* sequences. A network diagram of all haplotypes was constructed based on median joining calculations as implemented in PopARTv1.7^116,117^.

### Demographic history

The historical demographic expansion of each population was tested using Tajima’s D^63^ and Fu’s F^61^ statistics in ARELIQUIN v3.5^109^. In addition, Harpending’s raggedness index, *Hri*^64^ implemented in ARLEQUIN v3.5^109^ and the mismatch distribution^118,119^ implemented in DnaSp6^104^ were applied to find out whether the sampled populations were demographically stable or expanding or decreasing over time. Both tests were applied in order to detect whether the populations deviate from what would be expected from a sudden population expansion model. A significant *Hri* value (p < 0.05) rejects a model of sudden population expansion^112^.

The precise time for expansion was calculated using the equation τ = 2ut^118^; where t is the expansion time. 2u was calculated using the equation 2u = μk^120^, where μ is the mutation rate which is 1–2%/site/year for the mitochondrial *Cyt b* gene in fishes (Carangidae)^121–123^ and k is the number of nucleotides covered in the data (307 bp).

### Supplementary Information

Supplementary Information.

## Data Availability

All data generated or analyzed during this study is included in this published article (and its supplementary information files). In addition, the sequence data generated in this study is available in the NCBI-GenBank under the accession numbers: OR188721 to OR188758 and OR546246 to OR546278 (https://www.ncbi.nlm.nih.gov/genbank/).
