# Supplementary material for: Genetic analyses reveal panmixia in Indian waters and population subdivision across Indian Ocean and Indo-Malay Archipelago for Decapterus russelli
Source: Sci Rep. 2023 Dec 21;13:22860. doi: 10.1038/s41598-023-49805-8 (PMC10739887; doi:10.1038/s41598-023-49805-8)
Supplement: Supplementary file 1 — Supplementary Information. [file 41598_2023_49805_MOESM1_ESM.pdf]

Genetic analyses reveal panmixia in Indian waters and population subdivision across Indian Ocean and Indo-Malay Archipelago for *Decapterus russelli*

Anjaly Jose[12*], Sandhya Sukumaran[1], Subal Kumar Roul[1], Abdul Azeez P[1], Shoba Joe Kizhakudan[1], Neenu Raj[1], Nisha K[1] and A. Gopalakrishnan[1]

[1]Marine Biotechnology Fish Nutrition and Health Division, ICAR-Central Marine Fisheries Research Institute, Ernakulam North P O, Kochi, Kerala, India-682018
[2]Mangalore University, Mangalagangotri, Mangalore, Karnataka, India-574199

*Corresponding author

Email address: anjalyelizabethjose@gmail.com

Supplementary Table S1. Parameters of variability estimates for *D. russelli* populations from Indian Ocean based on mitochondrial *Cyt b.*

	Indian Ocean				
	East coast		West Coast		
	Chennai	Puri	Cochin	Mangalore	Veraval
Number of samples	25	25	25	25	25
Number of haplotypes	12	8	5	7	17
Number of polymorphic sites	10	10	4	26	25
Haplotype diversity, Hd	0.80667	0.66333	0.4233	0.43000	0.90333
Nucleotide diversity	0.00117	0.00124	0.00071	0.00231	0.00283
Tajimas D	-1.15753	-1.51406	0.00000	-2.30550	-1.51406
Fu's Fs	-1.06131	-2.12796	0.00000	0.91456	-2.12796
Hri index	0.71200	0.49861	0.00000	0.60492	0.49861

Supplementary Table S2: Population pairwise Fst (below diagonal) and genetic diversity (distance) (upper diagonal) of *D. russelli* inferred by mtDNA *Cyt b*. Bold number indicates significant value at $p < 0.05$. The genetic diversity (distance) values were presented in percentage (%) in the text.

Kelang	Carita	Labuan	Tambelan	Pekalongan	Kinabalu	Sandakan	Toli-Toli	Makassar	Arafura	Mangalore	Cochin	Veraval	Chennai	Puri	
0.00	0.00	0.00	0.00	0.00	0.00	0.00	0.01	0.01	0.00	0.01	0.01	0.01	0.01	0.01	Kelang
	0.00	0.00	0.00	0.00	0.00	0.00	0.01	0.01	0.00	0.01	0.01	0.01	0.01	0.01	Carita
		0.00	0.00	0.00	0.00	0.00	0.01	0.01	0.00	0.01	0.01	0.01	0.01	0.01	Labuan
			0.00	0.00	0.00	0.00	0.02	0.01	0.00	0.01	0.01	0.01	0.01	0.01	Tambelan
				0.00	0.00	0.00	0.01	0.01	0.00	0.01	0.01	0.01	0.01	0.01	Pekalongan
					0.00	0.00	0.01	0.01	0.00	0.01	0.01	0.01	0.01	0.01	Kinabalu
						0.00	0.01	0.01	0.00	0.01	0.01	0.01	0.01	0.01	Sandakan
							0.01	0.01	0.01	0.02	0.01	0.01	0.01	0.02	Toli-toli
								0.01	0.01	0.02	0.01	0.01	0.01	0.01	Makassar
									0.01	0.01	0.01	0.01	0.01	0.01	Arafura
										0.00	0.00	0.00	0.00	0.00	Mangalore
											0.00	0.00	0.00	0.00	Cochin
												0.00	0.00	0.00	Veraval
													0.00	0.00	Chennai
														0.00	Puri

Kelang	0.00000		
Carita	-0.02417	0.00000	
Labuan	-0.01510	-0.01204	0.00000

Tambelan	-0.00113	-0.00138	0.00924	0.00000											
Pekalongan	-0.01257	-0.00942	-0.00212	0.00040	0.00000										
Kinabalu	-0.00469	-0.00936	0.00238	-0.01922	-0.00488	0.00000									
Sandakan	-0.00840	-0.00529	0.00933	0.00223	0.00012	-0.00483	0.00000								
Toli-Toli	**0.61915**	**0.70654**	**0.73550**	**0.75131**	**0.76553**	**0.72390**	**0.77852**	0.00000							
Makassar	**0.52170**	**0.63874**	**0.67444**	**0.69585**	**0.71317**	**0.66085**	0.77852	-0.01827	0.00000						
Arafura	**0.15225**	**0.01065**	0.01267	0.00517	-0.00417	0.00889	**0.00778**	**0.78234**	**0.73624**	0.00000					
Mangalore	**0.80987**	**0.83334**	**0.82331**	**0.86986**	**0.87052**	**0.95780**	**0.88646**	**0.65484**	**0.60943**	**0.91962**	0.00000				
Cochin	**0.96443**	**0.91393**	**0.87237**	**0.94205**	**0.87052**	**0.95780**	**0.95005**	**0.70953**	**0.67552**	**1.00000**	0.00000	0.00000			
Veraval	**0.92972**	**0.89701**	**0.86239**	**0.92698**	**0.91826**	**0.93829**	**0.93686**	**0.69464**	**0.65790**	**0.98301**	0.00000	0.00000	0.00000		
Chennai	**0.94677**	**0.90538**	**0.86741**	**0.93445**	**0.92440**	**0.94794**	**0.94340**	**0.70397**	**0.66868**	**0.99143**	0.00000	0.00000	-0.02740	0.00000	
Puri	**0.92972**	**0.89701**	**0.86251**	**0.92698**	**0.91826**	**0.93829**	**0.93686**	**0.69850**	**0.66198**	**0.98301**	0.00000	0.00000	0.00000	0.00000	0.00000

Supplementary Table S3. Parameters of variability estimates for *D. russelli* populations from Indian Ocean based on nuclear *DrAldoB1.*

Indian Ocean					
East Coast			West Coast		
Locations	Chennai	Puri	Cochin	Mangalore	Veraval
Number of samples	50	50	50	50	50
Number of polymorphic sites	42	29	11	23	3
Number of haplotypes	12	5	8	9	3
Haplotype diversity	0.628	0.155	0.391	0.480	0.153
Nucleotide diversity	0.022	0.012	0.005	0.018	0.001

Supplementary Table S4. Population pairwise F_{ST} (below diagonal) and genetic diversity (upper diagonal) inferred by intron 1 of *Aldolase b.* Bold number indicates significant value at $p < 0.05$. The genetic diversity (distance) values were presented in percentage (%) in the text.

	Chennai	Puri	Cochin	Mangalore	Veraval	IMA	
	0.00	0.01	0.01	0.01	0.01	0.016	Chennai
		0.01	0.009	0.01	0.007	0.019	Puri
			0.00	0.01	0.003	0.015	Cochin
				0.01	0.01	0.014	Mangalore
					0.001	0.013	Veraval
						0.001	IMA
Chennai	0.00						
Puri	0.035	0.00					
Cochin	0.040	0.042	0.00				
Mangalore	0.050	0.052	0.083	0.00			
Veraval	0.058	0.035	0.030	0.090	0.00		
IMA	**0.760**	**0.621**	**0.125**	**0.572**	**0.611**		

Supplementary Table S5. Results of the analysis of molecular variance (AMOVA) for *D. russelli* showing F- statistics analysis for intron 1 of *Aldolase b*

Hierarchical level	F- statistics	P value
**Among IO**		
Among regions (F_{CT})	-0.001	ns
Among populations within region (F_{SC})	0.053	ns
Among individuals within populations (F_{ST})	0.052	ns
**Between IO and IMA**		
Among regions (F_{CT})	80.02	*
Among populations within region (F_{SC})	14.80	*
Among individuals within populations (F_{ST})	5.98	*

IMA: Indo-Malay Archipelago; IO: Indian Ocean.

ns, not significant (p>0.05)

*P<0.01

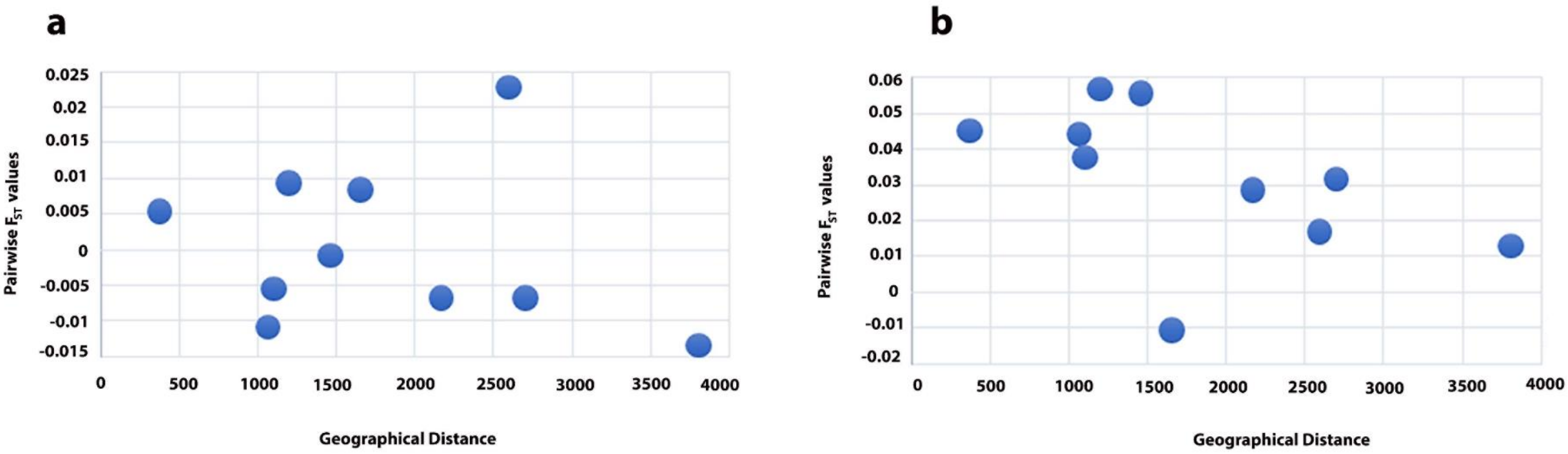

Supplementary Figure S1. Scatter plot of geographical distance vs pairwise F_{ST} value of *D. russelli* individuals based on (a) *Cyt b* (b) *Aldolase b* from Mantel test.

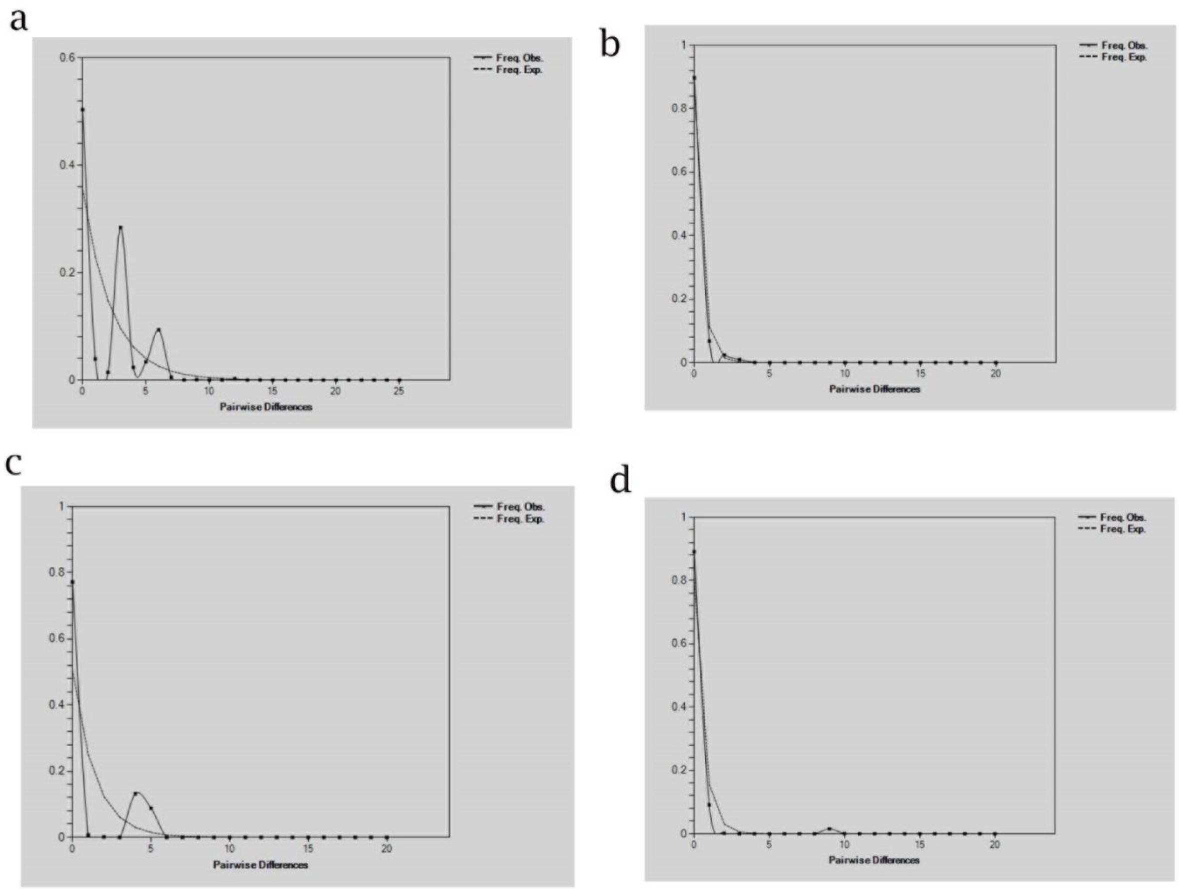

Supplementary Figure S2. Mismatch distribution (pairwise difference) for *D. russelli* for (a) whole population (b) IMA lineage 1 (c) IMA lineage 2 (d) IO lineage.

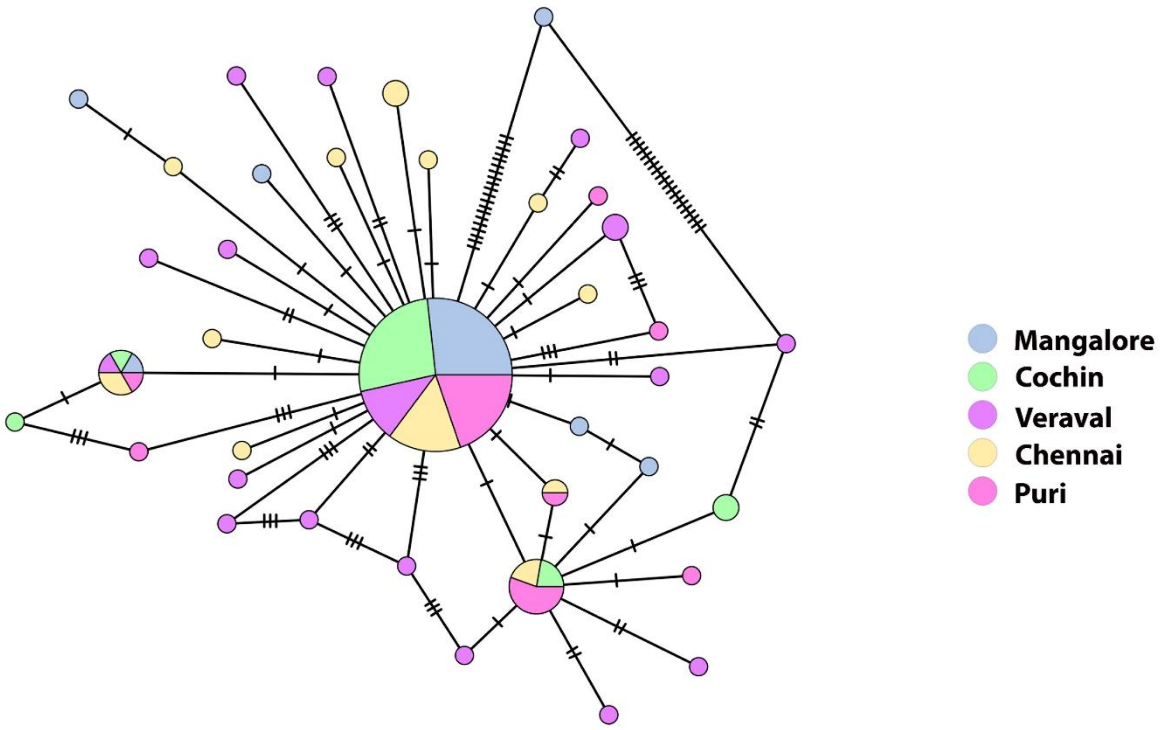

Supplementary Figure S3. Minimum spanning network (MSN) inferred from mtDNA *Cyt b*. Coloured close circles represents different regions (refer to legend)

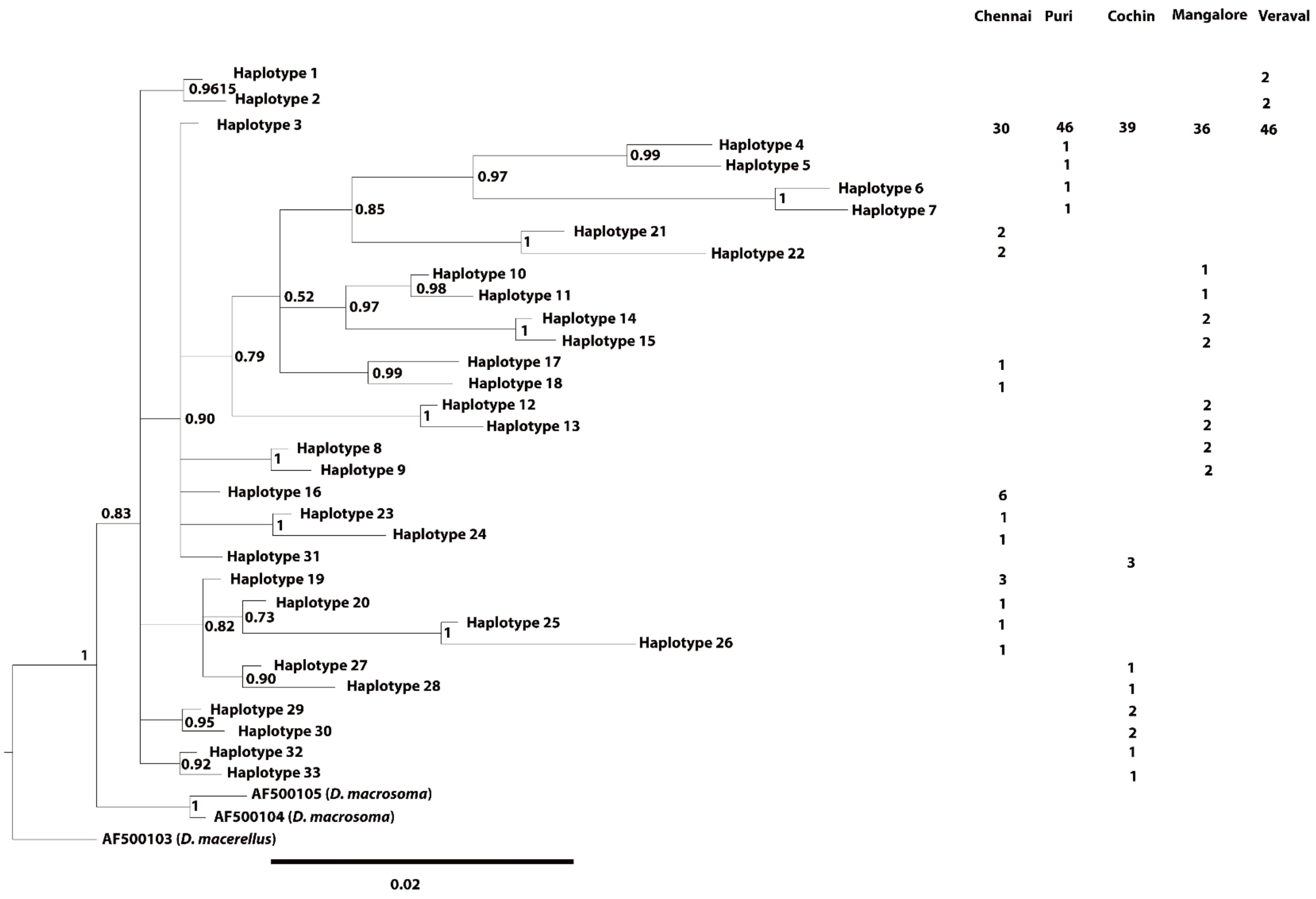

Supplementary Figure S4. Bayesian phylogenetic tree from the Intron 1 partial sequences of Aldolase b gene of *D. russelli* from Indian Ocean.

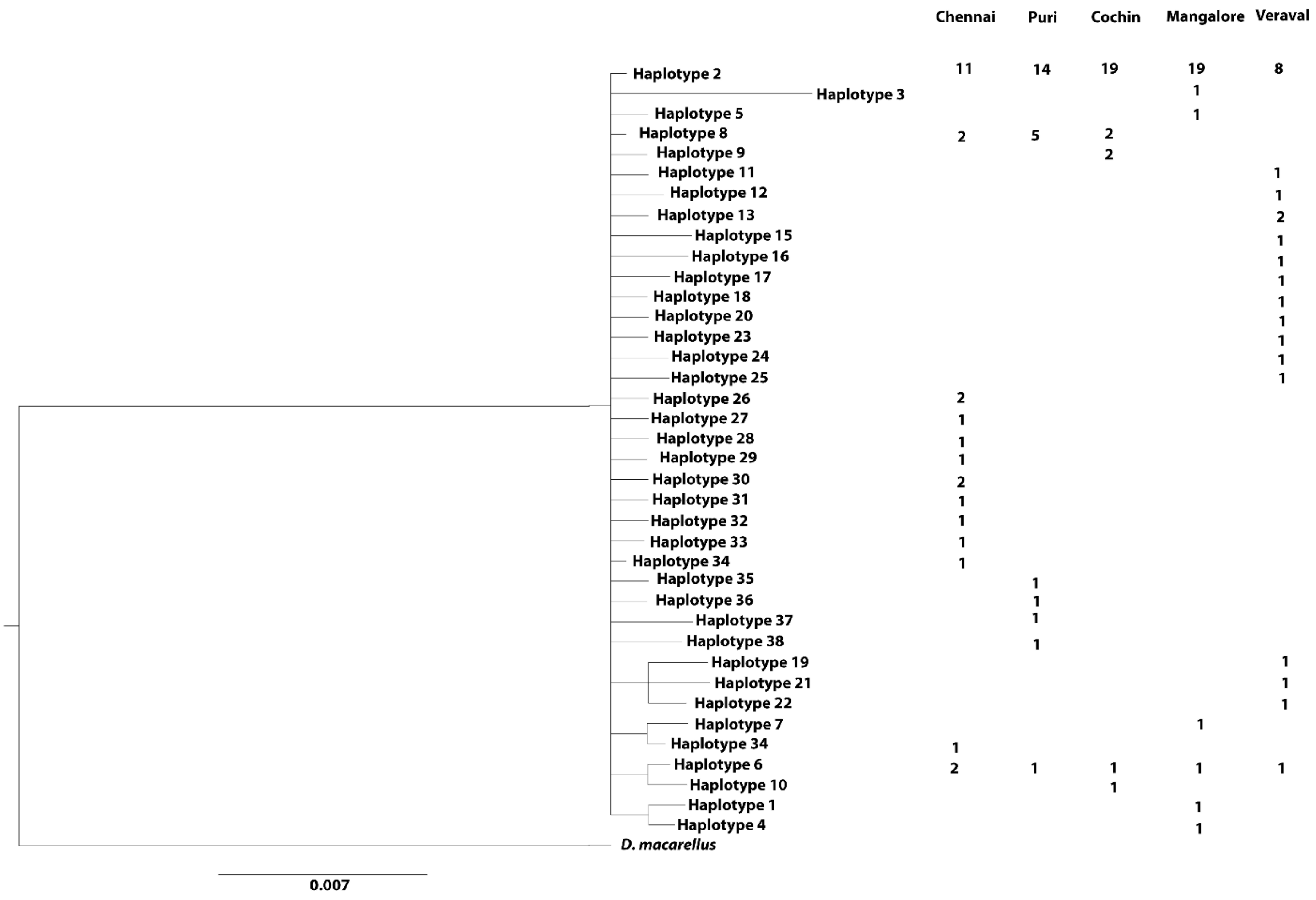

Supplementary Figure S5. Bayesian phylogenetic tree from the partial sequences of *Cyt b* gene of *D. russelli* from Indian Ocean.
